# Hepatic Hemangioma: Review of Imaging and Therapeutic Strategies

**DOI:** 10.3390/medicina60030449

**Published:** 2024-03-08

**Authors:** Arkadiusz Kacała, Mateusz Dorochowicz, Iwona Matus, Michał Puła, Adrian Korbecki, Michał Sobański, Jagoda Jacków-Nowicka, Dariusz Patrzałek, Dariusz Janczak, Maciej Guziński

**Affiliations:** 1 Department of General, Interventional and Neuroradiology, Wroclaw Medical University, 50-367 Wrocław, Poland; jagoda.jackow-nowicka@umw.edu.pl (J.J.-N.); maciej.guzinski@umw.edu.pl (M.G.); 2 Faculty of Medicine, Wroclaw Medical University, 50-367 Wrocław, Poland; m.dorochowicz97@gmail.com (M.D.); iwona.matus4@gmail.com (I.M.); 3 Department of General, Interventional and Neuroradiology, Wroclaw University Hospital, 50-556 Wrocław, Poland; michal.pula97@gmail.com (M.P.); lek.adriankorbecki@gmail.com (A.K.); michalsobanski11@wp.pl (M.S.); 4 Department of Vascular, General and Transplantation Surgery, Wroclaw Medical University, 50-367 Wrocław, Poland; dariusz.patrzalek@umw.edu.pl (D.P.); dariusz.janczak@umw.edu.pl (D.J.)

**Keywords:** hepatic hemangiomas, atypical hepatic hemangioma, computed tomography (CT), magnetic resonance (MR), ultrasound (US), contrast-enhanced ultrasound (CEUS), transarterial chemoembolization (TACE)

## Abstract

Hepatic hemangiomas are the most common benign liver tumors. Typically, small- to medium-sized hemangiomas are asymptomatic and discovered incidentally through the widespread use of imaging techniques. Giant hemangiomas (>5 cm) have a higher risk of complications. A variety of imaging methods are used for diagnosis. Cavernous hemangioma is the most frequent type, but radiologists must be aware of other varieties. Conservative management is often adequate, but some cases necessitate targeted interventions. Although surgery was traditionally the main treatment, the evolution of minimally invasive procedures now often recommends transarterial chemoembolization as the treatment of choice.

## 1. Introduction

Hepatic hemangiomas, the most prevalent benign liver tumors, are characterized as slow-flow venous malformations with an incidence rate ranging between 0.4% and 20.0% [1,2,3]. These tumors are primarily comprised of endothelial cells originating from the hepatic artery [4,5,6]. Cavernous hemangiomas represent the most frequent pathological subtype. Notably, there is a predilection for women, with reported female-to-male ratios reaching as high as 5:1 [7,8]. The vast majority of hepatic hemangiomas are asymptomatic, maintain a stable size, do not affect liver function, and are incidentally detected during routine abdominal imaging [9,10,11]. The detection of hepatic hemangiomas has significantly increased in recent years, largely due to advancements in imaging technologies. Hemangiomas that are small to medium in size, defined as less than 4 cm in diameter, typically remain asymptomatic and are managed conservatively.

Giant hemangiomas, particularly those that exhibit progressive growth, pose a higher risk of serious complications, including local compression effects due to the tumor’s volume, hemorrhage, Kasabach–Merritt Syndrome, or Budd–Chiari syndrome [12,13,14,15,16]. Such hemangiomas can reach up to 40 cm in diameter and are most commonly found in the right liver lobe, especially in segment IV [17].

This review explores recent developments in the literature on hepatic hemangiomas, with a focus on advancements in surgical and minimally invasive treatment modalities. The shift towards minimally invasive techniques has expanded the treatment options for hemangiomas previously considered inoperable, reflecting a significant evolution in therapeutic approaches.

## 2. Materials and Methods

### 2.1. Literature Search Strategy

A systematic literature search was executed across several electronic databases, including PubMed, MEDLINE, Embase, and Google Scholar, spanning from their inception to 12.01.2024, without imposing language constraints. Search terms employed encompassed “hepatic hemangioma”, “liver hemangioma”, “imaging”, “diagnosis”, “therapeutic strategies”, “management”, “embolization”, “surgery,” “radiotherapy”, “review”, and “treatment”, along with their various combinations.

### 2.2. Inclusion and Exclusion Criteria

Inclusion criteria were applied to articles that offered insights into imaging techniques for hepatic hemangiomas, diagnostic methodologies, and therapeutic interventions. Studies that discussed clinical manifestations, histopathological features, epidemiological data, and treatment outcomes were also considered. The selection was limited to peer-reviewed articles, review articles, case reports, and clinical trials. Exclusions were made for animal studies, conference abstracts, and publications without accessible full texts.

### 2.3. Data Extraction and Analysis

Initial screening involved two independent reviewers assessing the titles and abstracts for relevance. Subsequently, full-text articles were scrutinized to confirm their suitability against the predefined inclusion and exclusion criteria. Any disagreements between the initial reviewers were arbitrated by a third, independent reviewer. The data collation process focused on extracting information regarding the study’s design, participant demographics, sample size, imaging results, diagnostic approaches, therapeutic modalities, outcomes, and any reported complications.

### 2.4. Quality Assessment

The methodological quality of the included studies was evaluated using appropriate tools tailored to the study design: the Cochrane Risk of Bias Tool for randomized controlled trials and the Newcastle–Ottawa Scale for observational studies. Case reports and case series were assessed for their informational clarity and the pertinence of the data provided.

### 2.5. Synthesis of Results

A narrative synthesis was conducted on the data harvested from the selected studies, aiming to compile a comprehensive summary of the current knowledge on imaging modalities, the diagnostic complexities, and the array of therapeutic interventions available for managing hepatic hemangiomas.

## 3. Clinical Manifestations of Hepatic Hemangiomas

In the majority of instances, hepatic hemangiomas are asymptomatic and are fortuitously identified during imaging procedures conducted for unrelated medical conditions. This observation holds particularly true for hemangiomas of small to medium size. However, in the case of giant hemangiomas (exceeding 5 cm in diameter) that exhibit rapid growth, various symptoms may manifest, albeit these are typically nonspecific and mimic those associated with a range of other disorders, especially those of gastrointestinal origin. The clinical manifestations commonly observed in patients with giant hepatic hemangiomas include pain in the upper abdominal quadrants, nausea, abdominal distension, dyspepsia, and early satiety. Physical examination seldom reveals a palpable mass.

The complications associated with hepatic hemangiomas are predominantly contingent upon the lesion’s size and anatomical location. Mechanical complications can arise, such as spontaneous or trauma-induced rupture, and local compression effects on adjacent anatomical structures. For example, compression of the bile ducts can precipitate jaundice or hemobilia; impingement on the stomach may result in gastric obstruction, leading to symptoms of early satiety and dyspepsia; and compression of the hepatic veins by the tumor mass can obstruct venous outflow, culminating in Budd–Chiari syndrome. [16,18]. Inflammatory complications can manifest as either acute or chronic fever and pain. Hemorrhagic complications encompass intratumoral or intraperitoneal hemorrhage, which may occur with or without associated consumptive coagulopathy. One notable condition in this context is Kasabach–Merritt syndrome, predominantly observed in giant hepatic hemangiomas. This syndrome is characterized by thrombocytopenia, microangiopathic hemolytic anemia, and disseminated intravascular coagulation. Additionally, Klippel–Trenaunay syndrome, a form of congenital hemiatrophy, can lead to the development of nevus flammeus and hemimegalencephaly. Von Hippel–Lindau disease is another condition that results in the formation of hemangiomas in multiple organs, including the brain, retina, pancreas, and liver, further complicating the clinical picture [13,14,15]. While Kasabach–Merritt syndrome is predominantly reported in pediatric populations, there have been documented instances of its occurrence in adults presenting with giant hepatic hemangiomas [19,20]. It can manifest as a symptom of giant hemangioma, leading to consumption coagulopathy and ensuing thrombocytopenia, prolonged prothrombin time and partial thromboplastin time, and hypofibrinogenemia caused by endothelial defects within the hemangioma. These manifestations may occur alongside or without microangiopathic hemolytic anemia [21]. Degenerative complications include thrombosis, progressive fibrosis, and sclerosis. Noteworthy cases of hepatic hemangiomas include calcified, pedunculated (presents risk of torsion), hepatic hemangioma occurring on the steatotic or cirrhotic liver, and with accompanying arteriovenous shunt or heart failure.

## 4. Diagnostic Approaches for Hepatic Hemangiomas

Hepatic hemangiomas are commonly identified incidentally through cross-sectional abdominal imaging conducted for routine screening or for purposes unrelated to the investigation of a potential hepatic mass [9,10,11]. This incidental detection is attributable to the predominantly asymptomatic nature of these lesions, which frequently remain unnoticed during standard physical examinations.

The diagnosis of hepatic hemangiomas utilizes a spectrum of imaging modalities, including conventional ultrasound (US, including B-mode and Doppler), contrast-enhanced ultrasound (CEUS), contrast-enhanced computed tomography (CT), magnetic resonance imaging (MRI), angiography, and nuclear imaging (specifically, scintigraphic studies utilizing Technetium-99m-labeled red blood cells). The specificity and sensitivity of these diagnostic techniques are delineated in Table 1 [22].

These diagnostic modalities offer considerable specificity in differentiating hepatic hemangiomas from other vascular neoplasms, benign entities such as adenomas, or malignant lesions including hepatocellular carcinoma (HCC), metastases, and dysplastic nodules. Typically, hepatic hemangiomas are categorized into three histological subtypes: capillary hemangioma, cavernous hemangioma, and sclerosing hemangioma.

### 4.1. Ultrasound (US)

**Figure 1:**
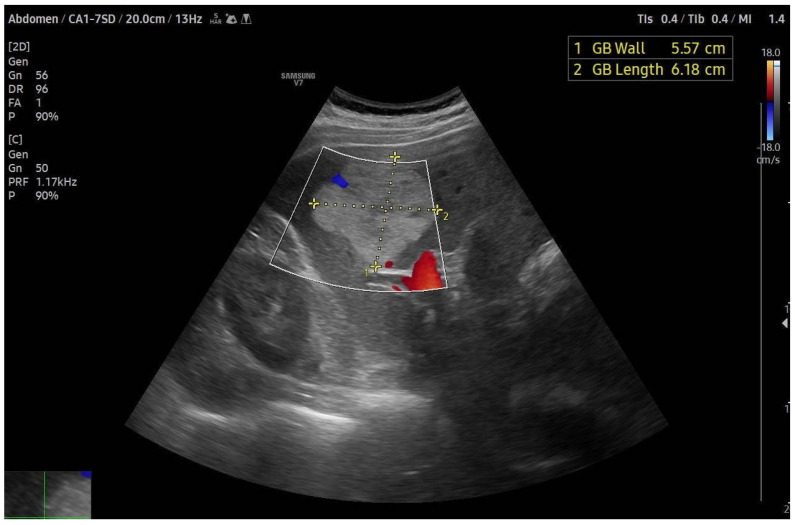
Slightly heterogeneous hyperechoic lesion with absence of flow on color Doppler, characteristic of a large hepatic hemangioma.

Ultrasound (US) frequently serves as the initial diagnostic modality for hepatic hemangiomas, favored for its wide availability, non-ionizing nature, and repeatability. However, a significant limitation of US is its dependency on the operator’s expertise and the patient’s specific characteristics, rendering it highly sensitive to both operator and patient factors. On greyscale ultrasound, hepatic hemangiomas typically present as hyperechoic, well-circumscribed lesions with a uniform appearance, or as hypoechoic masses featuring a hyperechoic rim [23,24,25,26] (Figure 1). The hyperechoic pattern observed in ultrasound images of hepatic hemangiomas is linked to their histological makeup, where the echogenicity results from the numerous interfaces between the endothelium-lined sinuses constituting the lesions and the encapsulated blood. Smaller hepatic hemangiomas commonly exhibit this hyperechoic characteristic. In contrast, larger lesions might show heterogeneity, characterized by mixed echogenicity (both hypo- and hyperechoic) arising from potential necrosis, hemorrhage, or fibrosis, leading to classification as atypical hepatic hemangiomas. Doppler ultrasound assessments of most hepatic hemangiomas reveal minimal to absent Doppler flow signals [27].

Nevertheless, it is imperative to approach the diagnosis of every hyperechoic mass with caution before categorizing it as a hepatic hemangioma. This echogenic pattern may also manifest in a spectrum of other hepatic conditions, encompassing both benign entities (e.g., adenomas) and malignant pathologies (such as hepatocellular carcinoma and metastatic lesions). Consistency in imaging findings across successive examinations constitutes a reliable marker for benign pathology in clinical settings. Ultrasound demonstrates high diagnostic accuracy in distinguishing hepatic hemangiomas from malignant hyperechoic masses, evidencing a sensitivity of 94.1% and specificity of 80.0% for lesions smaller than 3 cm in diameter. The lack of detectable blood flow within a lesion on Doppler ultrasound serves as a robust discriminant for differentiating hepatic hemangioma from hepatocellular carcinoma (HCC), the latter typically exhibiting intra- or peritumoral vascular signals [28].

In the context of hypoechoic lesions, the identification of a peripheral echogenic halo may indicate a hepatic hemangioma. Conversely, the presence of a hypoechoic rim encircling the lesion, often referred to as the “target sign”, is infrequently associated with hepatic hemangiomas [27]. Special caution is warranted in the evaluation of hepatic lesions within a steatotic liver, where the altered echotexture may cause a typically hyperechoic hemangioma to appear hypoechoic against the background of an intensely hyperechoic liver parenchyma.

### 4.2. Contrast-Enhanced Ultrasound (CEUS)

Contrast-enhanced ultrasound (CEUS) represents a more specific diagnostic modality for hepatic hemangiomas (HH) compared to traditional ultrasound techniques. By employing microbubble contrast agents that enhance the visualization of the microvasculature, CEUS facilitates real-time perfusion imaging that mirrors the vascular patterns observable in CT imaging. This feature is exceedingly beneficial for differentiating liver nodules and accurately identifying HH in contrast to adenomas, focal nodular hyperplasia (FNH), hepatocellular carcinoma (HCC), or metastatic lesions. Characteristically, a typical HH demonstrates peripheral nodular enhancement in the arterial phase, followed by complete (occasionally incomplete) centripetal filling in the portal venous and late phases. This enhancement pattern boasts a high sensitivity (98%) for the identification of histologically confirmed HH. Nonetheless, it is critical to acknowledge that HH may, albeit infrequently, exhibit a centrifugal enhancement pattern as well [29,30].

CEUS provides several significant advantages, including the capability for real-time examination and instantaneous results. It allows for the concurrent assessment of multiple lesions, offers the repeatability necessary for follow-up evaluations, and permits the re-injection of contrast agents for enhanced imaging [31]. However, the diagnostic accuracy of CEUS can be compromised in patients with steatosis (fatty liver) or for lesions deeply situated within the body. Moreover, imaging comprehensive views of a large hepatic hemangioma presents a challenge due to the limited penetration depth and field of view of the ultrasound probe [3].

### 4.3. Endoscopic Ultrasound (EUS)

Endoscopic ultrasound (EUS) offers the ability to visualize and biopsy small, solid liver lesions that may not be detectable through other imaging techniques, or that become evident only during routine staging for gastrointestinal cancers. However, the precise diagnostic utility of EUS in the context of liver diseases remains to be fully elucidated, highlighting the need for comparative studies to define its role more clearly. The therapeutic applications of EUS in hepatic management are expanding [32]. EUS facilitates guided interventions such as fine-needle aspiration (FNA) or biopsies when necessary. Although biopsy is typically not indicated for conventional hepatic hemangiomas, EUS-guided procedures can improve the accuracy and safety of biopsies in atypical cases or when the diagnosis is uncertain [33,34]. It is essential to acknowledge that EUS is a semi-invasive technique, carrying inherent risks of complications.

### 4.4. Computed Tomography

**Figure 2:**
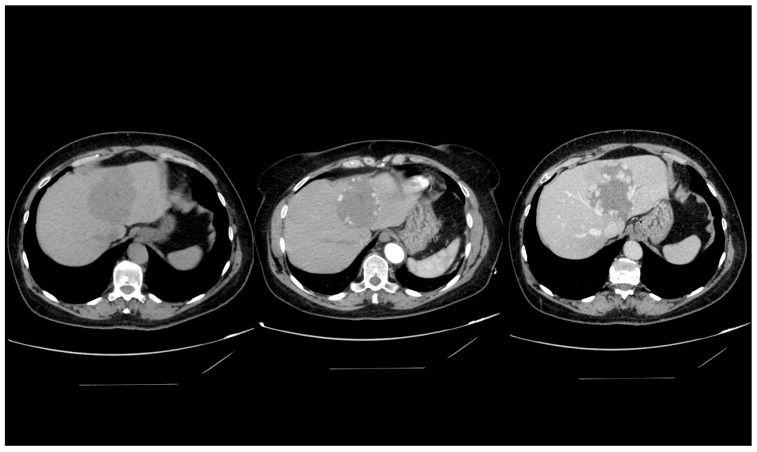
Computed tomography imaging of a giant hepatic hemangioma. The sequence illustrates a precontrast image on the left, an arterial phase image in the center, and a venous phase image on the right. This series effectively demonstrates the delayed contrast filling from the tumor’s periphery, characteristic of a hepatic hemangioma.

The characteristic imaging feature of a hepatic hemangioma on computed tomography (CT) scans is a well-circumscribed, hypodense lesion. Upon administration of contrast medium, it demonstrates peripheral nodular enhancement, followed by gradual and homogeneous centripetal fill-in (Figure 2). Nevertheless, this distinct enhancement pattern may not be discernible in lesions smaller than 5 mm, complicating their accurate identification. Atypical hepatic hemangiomas can present with a variety of enhancement patterns on CT imaging [31,35].

In the context of hepatic steatosis (fatty liver), particular caution is warranted, as a typical hemangioma might appear hyperdense in comparison to the surrounding hepatic parenchyma. Major limitations of CT imaging include the risk of radiation exposure and the use of iodine-based contrast materials, which carry a potential risk for allergic reactions or contrast-induced nephropathy.

### 4.5. Magnetic Resonance Imaging

**Figure 3:**
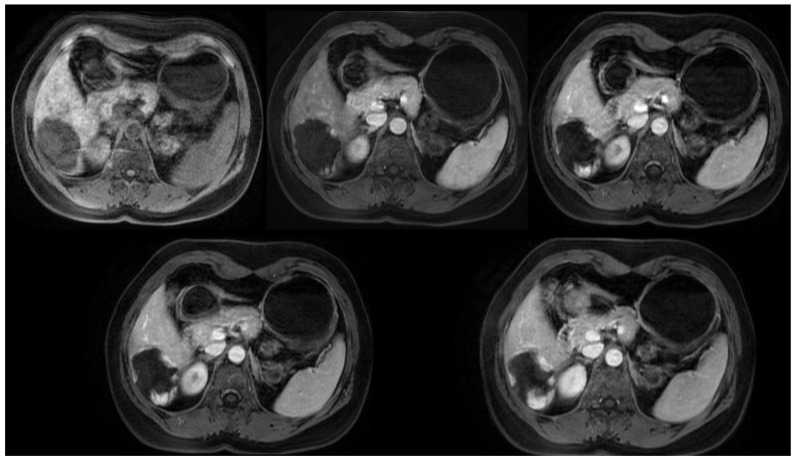
Axial T1-weighted MRI with contrast, multiphase study of a giant hepatic hemangioma. This image sequence demonstrates the gradual peripheral-to-central filling of the hemangioma, highlighting the characteristic enhancement pattern.

In magnetic resonance imaging (MRI), hepatic hemangiomas are typically characterized by a well-defined, homogenous morphology, manifesting as hypointense on T1-weighted sequences and hyperintense on T2-weighted sequences, a feature often described as the “cotton-wool” appearance [36] (Figure 3). The differentiation between malignancies and hepatic hemangiomas, both of which exhibit hyperintensity on T2-weighted images, can be facilitated by modulating the echo time (TE). While malignant lesions tend to exhibit a reduction in signal intensity, hepatic hemangiomas display an enhanced signal intensity [37].

In MRI diagnostics, the contrast agent employed is gadolinium-based (UCA), rendering it an appropriate option for individuals with allergies to iodinated contrast agents or those with renal insufficiency for whom CT imaging with iodine-based contrast is contraindicated [38].

### 4.6. Technetium-99m-Labeled Red Blood Cell Imaging

Technetium-99m-labeled red blood cell (Tc-99m RBC) scintigraphy is a noninvasive diagnostic technique offering high specificity for identifying hepatic hemangiomas. In Tc-99m RBC imaging, hepatic hemangiomas exhibit a distinctive perfusion and blood pool mismatch, characterized by diminished perfusion in early dynamic phases with a progressive increment in radiotracer uptake during blood pool phases. Initially, the lesion presents as “cold” or less active, transitioning to intense activity typically within 1–2 h post Tc-99m injection. The sensitivity of this modality is contingent upon the lesion’s size: it is 17–20% for lesions under 1 cm, increases to 65–80% for lesions between 1 and 2 cm, and reaches nearly 100% for those exceeding 2 cm in diameter. The specificity of Tc-99m-labeled RBC scintigraphy, particularly when enhanced with Single Photon Emission Computed Tomography (SPECT), maintains a rate of 100% across all lesion sizes [17].

However, the sensitivity of this technique is influenced by various factors, including lesion size and anatomical location. Its diagnostic yield significantly improves when integrated with SPECT imaging, yet remains limited for lesions smaller than 1 cm or those positioned in anatomically complex regions [39,40]. The presence of persistent red blood cell activity within the heart, inferior vena cava, and major intrahepatic vessels poses challenges in detecting small hepatic hemangiomas located proximal to these vascular structures in SPECT images [41].

Despite its diagnostic precision, Tc-99m-labeled RBC scintigraphy has been largely superseded as a primary tool for hepatic hemangioma diagnosis due to several drawbacks, such as limited availability, elevated costs, lengthy procedural times, radiation exposure, and the advent of more advanced imaging technologies.

## 5. Imaging Characterization of Hemangioma Subtypes

Accurate identification of the distinct histological subtypes of hepatic hemangiomas plays a crucial role in the comprehensive diagnostic evaluation. The primary subtypes recognized within hepatic hemangiomas include cavernous hemangioma, capillary hemangioma (also referred to as fast-filling hemangioma), and sclerosing hemangioma [42]. The principal criterion for this classification is the extent of fibrous tissue present within the body of the hemangioma [18]. It is essential to recognize that the unique histological compositions of these lesions may result in imaging appearances that diverge from the conventional semiotics associated with hepatic hemangiomas.

### 5.1. Cavernous Hemangioma

Cavernous hemangioma is closely aligned with the established radiological profile of hepatic hemangiomas. However, its histological architecture diverges slightly from the classical hepatic hemangioma phenotype. The defining distinction lies in the presence of larger vascular spaces coupled with a reduced quantity of connective tissue. Such a configuration is predominantly observed in lesions smaller than 3 cm in diameter, characterized by well-defined margins and round or lobulated peripheries. Sonographically, this subtype manifests as a hyperechoic lesion with posterior acoustic enhancement, reflecting its histological composition [43]. Analogous to typical hepatic hemangiomas, cavernous hemangiomas seldom generate Doppler signals in both color-coded and spectral Doppler examinations [44]. The CT imaging of cavernous hemangiomas is consistent with the descriptions provided for hepatic hemangiomas in the ‘Computed Tomography’ section. However, MRI is the preferred imaging modality for cavernous hemangiomas, offering enhanced differentiation between hepatic hemangiomas and malignant hepatic tumors or cysts, contingent upon the inclusion of comprehensive imaging sequences. On T1-weighted MRI, cavernous hemangiomas present as masses with low signal intensity. T2-weighted and diffusion-weighted imaging (DWI) reveal homogeneous hyperintensity, frequently described as the “light bulb sign” [45], attributed to the lesion’s cavernous vascular structure facilitating slow blood flow and unrestricted water diffusion. The identification of this sign is particularly crucial in distinguishing flash-filling or sclerosing hemangiomas, which lack nodular enhancement. A T2 relaxation time threshold of 112 ms has demonstrated over 92% accuracy in differentiating hepatic hemangiomas from metastatic lesions [36]. In contrast-enhanced studies, cavernous hemangiomas exhibit enhancement patterns similar to those observed in CT, with early peripheral nodular enhancement followed by delayed, centripetal, and complete enhancement in later phases.

### 5.2. Capillary Hemangioma

Capillary hemangioma, also known as flash-filling or rapidly-filling hemangioma, represents the second histological subtype of hepatic hemangiomas, accounting for approximately 16% of all hepatic hemangiomas. This subtype is notably more prevalent in hemangiomas measuring less than 1 cm in diameter, comprising 42% of such cases [46]. Flash-filling hemangiomas pose a diagnostic challenge due to their similarity with numerous hypervascular tumors; they exhibit rapid, intense, and uniform contrast enhancement during the arterial phase of contrast-enhanced computed tomography (CT) and T2-weighted magnetic resonance imaging (MRI). A definitive diagnosis can be attained through delayed-phase CT or MRI, where vascular malformations continue to appear significantly attenuated or hyperintense, a characteristic not shared by hypervascular metastases [46]. In contrast to cavernous hemangiomas, capillary hemangiomas present as hypoechoic on ultrasound examinations due to their rapid blood flow through limited vessels and a fibrous stroma. Additionally, the application of color-coded Doppler ultrasound facilitates the detection of intralesional blood flow [42], further aiding in the differentiation of capillary hemangiomas from other vascular abnormalities.

### 5.3. Sclerosing Hemangioma

Sclerosing hemangioma, occasionally conceptualized as the involutive phase of hemangioma development, is alternatively known as thrombosed or hyalinized hemangioma. This subtype is relatively rare and seldom manifests clinically [46]. The process of hyalinization typically initiates at the lesion’s core, leading to the obliteration of vascular channels. Such pronounced alterations significantly modify the lesion’s radiological signature, complicating initial diagnostic efforts. Sclerotic transformation tends to produce a heterogenous imaging appearance, characterized by a central fibrous patch surrounded by cystic, fibrotic, and hemorrhagic zones [42]. These areas are distinctly visible as hypoechoic zones on ultrasound and hypodense regions on computed tomography (CT) scans. Furthermore, sclerosing hemangiomas frequently present with irregular contours, capsular retraction, and progressive volume reduction.

Contrary to the imaging profiles of other hepatic hemangioma subtypes, which are marked by pronounced hyperintensity on T2-weighted magnetic resonance (MR) images, hyalinized hemangiomas only demonstrate mild signal elevation. The absence of early enhancement and modest peripheral enhancement during late-phase MR imaging further delineates hyalinized hemangioma from conventional subtypes. Despite these distinctive imaging features, the radiological characteristics of sclerosing hemangioma may not suffice for a conclusive diagnosis, necessitating histopathological evaluation to exclude malignant entities [43].

## 6. Atypical Hepatic Hemangiomas in Imaging

### 6.1. Giant Haemangioma

The designation of a hemangioma as “giant” is subject to slight variations across medical literature, but it is commonly defined as a lesion measuring 5 cm in diameter or larger [4,46]. Features such as cystic cavities or central calcifications may be observed, with internal fibrotic septa being a frequent finding.

In ultrasonography (US), giant hemangiomas present a heterogeneous appearance. On non-contrast-enhanced computed tomography (CT) scans, they display a heterogeneous hypodense profile, which may include hypodense central regions [42,43,44,45,47,48,49]. Magnetic resonance imaging (MRI) reveals giant hepatic hemangiomas as hypointense on T1-weighted sequences, with potential alteration in the hyperintensity on T2-weighted images due to hypointense central zones [42,43,44]. Upon contrast administration, giant hepatic hemangiomas demonstrate peripheral globular enhancement and progressive centripetal filling, a hallmark pattern for these lesions. However, it is noteworthy that complete filling within the lesion is typically not achieved [50].

### 6.2. Hemangioma with Arterioportal Shunt

The co-occurrence of a hemangioma and an arterioportal shunt has been documented with an incidence rate of up to 26% [42,46,51,52]. This phenomenon is particularly prevalent in small capillary hemangiomas (<2 cm), where the high flow within the compact vascular spaces likely facilitates shunting through potential connections between the hepatic artery and the portal vein [43,52]. Arterioportal shunts are generally diminutive and frequently present as wedge-shaped or irregularly contoured transient enhancements in the arterial phase, indicative of a transient hepatic attenuation difference on computed tomography (CT) or a transient hepatic intensity difference on magnetic resonance imaging (MRI) [51,53,54,55]. Early opacification of adjacent portal vein branches post-contrast injection may signal the existence of an arterioportal shunt [52].

### 6.3. Hemangiomatosis

Hepatic hemangiomatosis is an uncommon disorder characterized by the proliferation of numerous hepatic hemangiomas dispersed throughout the liver parenchyma. Although typically asymptomatic in adults, hemangiomatosis is more commonly observed in newborns, where it can be associated with congestive heart failure [56,57]. Unlike the presentation of multiple discrete hepatic hemangiomas, hemangiomatosis features lesions that are ill-defined, extensive, and confluent, potentially encompassing the majority of the hepatic parenchyma. On sonography, these lesions may present as hypo- or hyperechoic, without the distinct peripheral globular enhancement seen in contrast-enhanced computed tomography (CT) and magnetic resonance imaging (MRI), complicating their differentiation from adjacent hepatic tissue. However, MRI retains its diagnostic utility in these cases, as T1- and T2-weighted sequences reveal distinctive signal patterns. Despite heterogeneous enhancement in the arterial phase, these lesions demonstrate progressive enhancement in dynamic late phases, maintaining typical signal intensities on T1- and T2-weighted MR sequences [43,46,56,57].

### 6.4. Pedunculated Haemangioma

The pedunculated hemangioma represents an exceptionally rare type of lesion that projects from the liver. It is characterized by a clear, encapsulated form, and is connected to the liver via a slender pedicle. Nonetheless, this pedicle may not always be discernible in axial imaging planes, making CT or MRI multiplanar reconstructions beneficial for confirming the hepatic origin of the mass. A specific complication associated with pedunculated hemangioma (PH) is volvulus, which occurs when the lesion twists around its pedicle. This can present as an acute abdominal condition, further complicated by necrosis or hemorrhage [43,58].

### 6.5. Hepatic Steatosis

Severe hepatic steatosis can influence the apparent enhancement patterns observed in focal hepatic lesions. Even lesions that are typically hypovascular, such as metastases, may demonstrate relatively high attenuation on computed tomography (CT), potentially mimicking hemangiomas with their persistent enhancement pattern. On ultrasound (US), hepatic hemangiomas generally appear as isoechoic or, more commonly, hypoechoic relative to the surrounding hyperintense steatotic liver tissue, frequently exhibiting posterior acoustic enhancement. Additionally, hemangiomas may present with a hypoechoic perilesional halo, a characteristic also observed in malignant tumors within a steatotic liver context. This atypical presentation often necessitates further evaluation through CT or magnetic resonance imaging (MRI). Although hemangiomas in a fatty liver might display a distinct halo on CT or MRI, accurate diagnosis is typically straightforward, facilitated by the lesions’ characteristic dynamic enhancement pattern. Magnetic resonance imaging is particularly advantageous for assessing hepatic hemangiomas (HHs) in the context of fatty liver, as the lesions’ hyperintensity on T2-weighted images is not affected by hepatic steatosis, maintaining its diagnostic utility [59,60,61].

### 6.6. Liver Cirrhosis

The detection and characterization of hepatic hemangiomas in the context of liver cirrhosis can be challenging, as these lesions tend to become more fibrous and decrease in size [59,62]. The prevalence of hepatic hemangiomas is lower in cirrhotic livers than in non-cirrhotic ones [63]. While hepatic hemangiomas may retain their characteristic imaging features, in advanced stages of cirrhosis, they often lose these distinctive traits, complicating diagnostic efforts [63,64]. Magnetic resonance imaging (MRI) is regarded as the preferred modality for evaluating hepatic hemangiomas due to its superior contrast resolution. The diagnostic sensitivity of MRI is further enhanced by T2-weighted sequences. In ultrasonography (US), both dysplastic nodules and hepatocellular carcinoma may appear as hyperechoic nodules, which can mimic the sonographic appearance of hepatic hemangiomas [64].

## 7. Histology Sampling

Given the vascular nature of hepatic hemangiomas, biopsy procedures involving histological sampling carry a considerable risk of hemorrhage, especially in the context of large, subcapsular lesions. Such procedures may result in severe complications, including mortality [65]. Additionally, the diagnostic yield of biopsy in this context is relatively low [66] leading to the recommendation that biopsy be reserved for lesions exhibiting atypical features.

## 8. Treatment

The vast majority of hepatic hemangiomas are characterized as small, asymptomatic, and exhibit stable dimensions, with patients generally maintaining normal liver function. These lesions are often incidentally identified during routine abdominal cross-sectional imaging studies [9,10,11]. Conservative management, encompassing periodic observation and surveillance via imaging at intervals of 6 or 12 months, is typically recommended as a suitable treatment strategy for these lesions. Notably, no cases of malignant transformation within hepatic hemangiomas have been documented [67,68]. Individuals presenting with new-onset pain, showing unresponsiveness to analgesics, undergoing estrogen therapy, experiencing pregnancy, or possessing large hepatic hemangiomas are advised to undergo extended observation as a component of their clinical management.

In instances where patients present with large lesions (exceeding 5 cm in diameter) that demonstrate progressive enlargement and are associated with symptomatology attributable to the lesions, specific therapeutic interventions become imperative [69]. It is essential to exclude all alternative etiologies for the symptoms, such as gastroesophageal reflux disease, peptic ulcer disease, or cholelithiasis, before contemplating interventional procedures.

Historically, surgical interventions, including resection, lobectomy, or enucleation, executed via open surgery or laparoscopy, were the preferred modalities for managing symptomatic cases [70,71]. However, with advancements in minimally invasive interventional techniques, surgical methods are no longer the first-line treatment for patients with multiple or extensive lesions. Alternative therapeutic options, such as radiofrequency ablation, microwave ablation, and arterial embolization, are now available for the treatment of symptomatic hepatic hemangiomas [72].

### 8.1. Surgical Approach

Surgical interventions for hepatic hemangiomas encompass segmental resection, lobectomy, or enucleation, with the selection of the technique being contingent upon the hemangioma’s size and location, alongside the surgeon’s expertise and preference. Enucleation is particularly advantageous for hemangiomas that are superficially located with a discernible plane on the liver surface, a zone characterized by compressed hepatic tissue with minimal vasculature due to the hemangioma’s expansion. This plane facilitates the hemangioma’s removal with negligible blood loss by separating the tumor capsule from the liver parenchyma, thus preserving the majority of the surrounding healthy liver tissue.

Conversely, hemangiomas that are deeply embedded within the liver parenchyma, lack an accessible surface from the Glisson capsule, or span an entire lobe necessitate hepatic resection as the optimal treatment strategy [73]. Surgical treatments, however, carry heightened risks of complications such as hemorrhage, infection, and increased financial costs, particularly for lesions exceeding 10 cm in diameter [74]. Furthermore, in instances of multiple hemangiomas or those proximal to the hepatic portal vessels, surgical resection can lead to less favorable outcomes and a pronounced risk of hemorrhage [75]. The feasibility of surgical intervention may also be limited by the lesion’s substantial size, its unfavorable positioning, or specific patient-related factors.

### 8.2. Radiofrequency Ablation

Radiofrequency ablation (RFA) represents a minimally invasive, efficacious modality for the treatment of both primary and metastatic hepatic neoplasms, with the procedure being executable via percutaneous or laparoscopic approaches [14,76]. Recent applications of percutaneous RFA have also demonstrated success in the management of liver hemangiomas [77,78,79]. The mechanism of RFA is postulated to entail the induction of localized thermal damage to the flat endothelial cells lining the extensively dilated, non-anastomotic vascular spaces characteristic of these tumors. The utility of RFA in this context is underpinned by the benign and hypervascular nature of liver hemangiomas.

This technique offers several advantages. Primarily, the benign character of the tumor obviates the need for excising a margin of healthy liver tissue surrounding the lesion. Additionally, the tumor’s composition, predominantly blood-filled cavities, facilitates the conspicuous collapse of the tumor tissue adjacent to the ablation zone upon application of radiofrequency energy. Moreover, the benign nature of any residual tumor post initial treatment negates the urgency for immediate follow-up intervention, given its non-progressive and non-metastasizing behavior [80].

Conversely, a notable drawback of RFA is the likelihood of hemolysis attributable to the vascular supply of the tumor. The magnitude and risk of this complication escalate with the tumor size, potentially culminating in a spectrum of conditions including hemoglobinuria, hemolytic jaundice, anemia, or renal impairment, contingent on the complication’s severity. Complication rates for tumors exceeding 10 cm in diameter have been reported between 34% and 100%, rendering RFA less suitable for managing giant hepatic hemangiomas [81,82].

### 8.3. Transarterial Embolization and Chemoembolization

In recent years, transarterial embolization (TAE) has been recognized as an effective strategy for the management of hepatic hemangiomas, functioning by obstructing the blood supply to the lesion without the use of chemotherapeutic agents. This technique is executed via an endovascular route, establishing itself as a cornerstone procedure for vascular occlusion. Similarly, transcatheter arterial chemoembolization (TACE) also aims to occlude the blood supply to the target lesion but incorporates an active biological agent alongside the embolization material. During TACE, various chemotherapeutic agents such as bleomycin, pingyangmycin, or ethanol, mixed with lipiodol, are utilized.

Despite the growing application of these methods, consensus regarding the efficacy of TAE in treating hemangiomas and the spectrum of potential complications remains elusive [74,83,84,85,86,87,88]. Liu et al. reported that TACE employing pingyangmycin yielded unsatisfactory outcomes in liver hemangioma treatments, highlighting a considerable risk of severe complications [85]. In contrast, Torkian et al., through a systematic review and meta-analysis, posited that TACE, when combined with agents like bleomycin, pingyangmycin, or ethanol mixed with lipiodol, was both safe and efficacious [74]. The popularity of TACE as a primary treatment for giant hepatic hemangiomas has surged. Li et al. documented a multi-center study involving 836 cases, where patients with giant hepatic hemangiomas underwent TACE using a pingyangmycin–lipiodol emulsion. The study reported no mortalities and only two instances of hepatic abscess as severe complications, alongside a notable decrease in lesion size, with the mean diameter reducing from 9.6 ± 0.8 cm to 3.6 ± 0.5 cm [6]. Yuan et al. evaluated the medium and long-term outcomes of TACE with a lipiodol–bleomycin emulsion in 241 patients, observing no mortalities or serious complications post-procedure. Patients experienced significant symptomatic relief, with no recurrence of symptoms during follow-up. A satisfactory tumor reduction rate, defined as a decrease in the lesion’s maximum diameter by more than 50%, was achieved in 88.1% of cases at the 6-month post-procedure mark [89].

The nature of the blood supply to hemangiomas significantly influences treatment outcomes and the incidence of complications. Therefore, in formulating treatment plans, clinicians should prioritize the evaluation of the hemangioma’s blood supply characteristics and dimensions. The current guidelines proposed by Ouyang et al. [90] and Zeng et al. [91] suggest specific considerations for assessing the blood supply type to hemangiomas (Table 2), indicating that in instances where the portal vein supplies blood, no abnormalities are detected in arterial and parenchymal phases, but portal venograms reveal abnormal blood-filled sinuses.

Postembolization syndrome (PES) represents the most frequent complication following transcatheter arterial chemoembolization (TACE), manifesting as influenza-like symptoms shortly after the intervention. The syndrome is predominantly characterized by abdominal pain, fever, nausea, and vomiting [92,93]. Kacała et al. observed PES in 45.7% of patients post-TACE, with the severity of symptoms varying across cases. In the majority of these instances, the administration of paracetamol was effective for pain management, and PES resolved spontaneously in all cases [93]. Basile et al. have posited that PES should be considered an expected outcome of TACE [94].

### 8.4. Liver Transplantation

Liver transplantation has been identified as a feasible therapeutic option for managing extensive hepatic hemangiomas associated with life-threatening coagulopathies, such as Kasabach–Merritt syndrome [95], among other indications [96]. Nevertheless, liver transplantation is regarded as a treatment of last resort due to its significant risks and the limited circumstances under which it is deemed appropriate.

## 9. Future Prospects

With the enhancement of imaging techniques such as MRI, CT, and ultrasound, diagnosing and monitoring hepatic hemangiomas will become significantly more straightforward. These advancements will facilitate the determination of tumor size, location, and characteristics, thereby guiding treatment decisions. Furthermore, the improvement in imaging methods is likely to lead to an increase in incidental findings. Although surgical management was once the preferred method for symptomatic hemangiomas, the rapid development of minimally invasive treatment options has led to a worldwide shift towards these alternatives. Future improvements in these techniques are expected to result in safer and more effective treatments, with lower risks and shorter recovery periods. Additional research is necessary to deepen our understanding of the natural progression of hepatic hemangiomas and to identify risk factors linked to their growth or complications. This knowledge will enable healthcare providers to make more informed decisions about monitoring and treatment. In summary, the outlook for patients with hepatic hemangiomas is optimistic, thanks to progress in diagnostic methods, treatment approaches, and research, all of which contribute to better patient outcomes for these benign liver tumors.

## 10. Discussion

Hepatic hemangiomas, representing the predominant benign mesenchymal neoplasms of the hepatic tissue, frequently manifest as asymptomatic entities, identified serendipitously through imaging modalities conducted for unrelated reasons, and seldom compromise hepatic functionality. From a histopathological perspective, these neoplasms are characterized by the presence of cavernous venous spaces, which are delineated by a lining of vascular endothelial cells and interspersed with connective tissue septa. The hemodynamics within these lesions are notably impaired, exhibiting a markedly reduced flow rate, with the hepatic artery serving as the principal source of vascular supply [97].

The lesions frequently exhibit stability in dimensional parameters and do not necessitate intervention beyond conservative management and vigilant observation. The lesions frequently exhibit stability in dimensional parameters and do not necessitate intervention beyond conservative management and vigilant observation [9,10,11]. There are no documented cases of hepatic hemangiomas undergoing malignant transformation; however, steroid or estrogen therapy, as well as pregnancy, have been observed to contribute to an increase in hemangioma size [67,68]. Giant hepatic hemangiomas may manifest with periodic pain, a sensation of abdominal fullness, or the detection of an upper abdominal mass, potentially leading to severe complications such as local compression, persistent pain, and serious conditions like obstructive jaundice, Kasabach–Merritt syndrome, Budd–Chiari syndrome, or spontaneous rupture resulting in intra-abdominal hemorrhage, with mortality rates reaching up to 70% [16,98,99]. The incidence of spontaneous rupture in hepatic hemangiomas ranges from 1% to 4%, with giant subcapsular lesions considered at higher risk [100,101,102,103].

Hepatic hemangiomas are commonly identified incidentally during imaging studies, yet their diagnosis and management continue to be debated among clinicians. Imaging techniques such as ultrasound (US), computed tomography (CT), magnetic resonance imaging (MRI), and angiography are crucial for diagnosing and characterizing hepatic hemangiomas. US is often the initial imaging technique employed due to its accessibility and cost-effectiveness. Despite this, there is no consensus on the definitive standard for diagnosing hepatic hemangioma. Historically, angiography was considered the gold standard for this purpose [104]. However, advancements and increased accessibility of various imaging methods have shifted this paradigm. Some researchers now view MRI as the gold standard for diagnosing hepatic hemangiomas [105,106], while others advocate for IV contrast-enhanced abdominal CT scans [69,107]. Furthermore, the role of biopsy and histopathological examination in evaluating liver lesions, especially atypical ones that may resemble malignancies, is crucial, despite the associated risks of such invasive procedures [108,109].

The management strategy for hepatic hemangiomas depends on various factors, including the size, location, symptoms, and potential complications of the lesion. Asymptomatic and small lesions often do not require intervention and can be monitored conservatively with regular imaging. Symptoms of enlarged hepatic hemangiomas are non-specific and can overlap with other pathological conditions; thus, alternative causes of abdominal symptoms such as gallstones, gastroesophageal reflux disease, or peptic ulcer disease should be excluded before considering interventional procedures. Surgery has traditionally been the preferred treatment for symptomatic patients or those with significant lesion growth [70,71]. When symptoms emerge, the lesion grows rapidly, or there is an increased risk of rupture, alternative therapeutic options become necessary, moving beyond the simple dichotomy of resection versus observation [98,110,111,112].

Surgical intervention necessitates careful consideration due to the potential for complications. Despite some evidence supporting surgical approaches, the risk of extended hospitalization, significant perioperative blood loss, and complications in lesions larger than 10 cm must be evaluated [74,113]. Alternatives to surgical and conservative treatments, such as radiofrequency ablation (RFA) and microwave ablation (MWA), have been explored but found lacking in efficacy and associated with complications, especially in giant lesions [79,81,82,114,115].

Transcatheter arterial chemoembolization (TACE) has recently gained traction as an effective treatment for hepatic hemangiomas. The efficacy of TACE has been extensively reviewed [6,88,89,93,116]. Bleomycin is known for its cytotoxic, antiangiogenic, and sclerosing properties, leading to DNA degradation and eliciting a generalized inflammatory response in the vicinity of the lesion and the portal area [117]. When combined with lipiodol, bleomycin’s embolic effect is enhanced, facilitating improved distribution of the chemotherapeutic agent to the targeted site.

However, it is crucial to recognize the limitations and potential complications inherent to each therapeutic approach. Interventional procedures, while effective in alleviating symptoms and reducing the size of hepatic hemangiomas, are associated with risks, including post-procedural bleeding, infection, and hepatic dysfunction. Consequently, a comprehensive evaluation of the risks and benefits of each treatment option is imperative, taking into account the unique characteristics and preferences of each patient.

In conclusion, the management of hepatic hemangiomas necessitates a multidisciplinary strategy, incorporating the expertise of radiologists, hepatologists, and surgeons to customize treatment plans according to the individual requirements of patients. Ongoing research efforts to enhance imaging methodologies and therapeutic techniques are essential to advance the management of hepatic hemangiomas and elevate patient care outcomes.

## 11. Conclusions

Hepatic cavernous hemangioma is the most prevalent type of benign liver tumor. When treatment is necessary for liver hemangiomas, surgical approaches like hepatic resection or enucleation, performed through open, laparoscopic, or robotic methods, have been historically deemed the first choice. However, in recent years, alternative therapies such as liver transplantation, radiofrequency ablation, transarterial embolization, and transarterial chemoembolization have also been gaining in importance. When deciding on the best treatment approach, it is essential to conduct a thorough assessment that takes into account various factors such as symptoms, size, location, and the presence of any coexisting medical conditions.

## Figures and Tables

**Table 1:** Sensitivity and specificity of diagnostic methods in hepatic hemangiomas.

Diagnostic Method	Sensitivity (%)	Specificity (%)
Ultrasonography	96.9	60.3
Computed tomography	98.3	55
Magnetic resonance imaging	100	85.7
Tc-99m RBC blood pool scintigraphy	67	100

**Table 2:** Characteristics of blood supply to hepatic hemangioma.

Type of Blood Supply	Artery Characteristics	Arterial Phase	Parenchymal Phase
Rich	Mild to moderate thickening of the arteries	Abnormal blood sinusoids	Dilatation of most blood sinusoids
Moderate	Mild thickening of the arteries	Abnormal blood sinusoids	Dilatation of some blood sinusoids
Poor	No thickening of the arteries	Very few abnormal blood sinusoids	No visible dilatation of blood sinusoids

## Data Availability

No new data were created or analyzed in this study. Data sharing is not applicable to this article.
